# Effects of the Fungal Bioherbicide, *Alternaria cassia* on Peroxidase, Pectinolytic and Proteolytic Activities in Sicklepod Seedlings

**DOI:** 10.3390/jof7121032

**Published:** 2021-11-30

**Authors:** Robert E. Hoagland, Clyde Douglas Boyette

**Affiliations:** 1 Crop Production Systems Research Unit, Department of Agriculture-Agricultural Research Service, Stoneville, MS 38776, USA; 2 Biological Control of Pests Research Unit, Department of Agriculture-Agricultural Research Service, Stoneville, MS 38776, USA; Doug.boyette@usda.gov

**Keywords:** *Senna obtusifolia*, *Alternaria cassiae*, fungal bioherbicide, biological weed control, sicklepod, plant defense, hydrolytic enzymes, peroxidase

## Abstract

Certain plant pathogens have demonstrated potential for use as bioherbicides for weed control, and numerous studies have been published on this subject for several decades. One of the early examples of an important fungal bioherbicide is *Alternaria cassiae*, isolated from the weed sicklepod (*Senna obtusifolia*). To gain further insight into biochemical interactions of this fungus and its host weed, we examined the effects of this bioherbicide on various enzymes associated with plant defense. Young sicklepod seedlings were challenged with *A. cassiae* spore inoculum and enzyme activities associated with plant defense (peroxidase, proteolytic, and pectinolytic) were assayed periodically over a 96-h time course on plants grown in continuous darkness or continuous light. Peroxidase activity increased with time in untreated control seedlings in both light and dark, but the effect was greater in the light. In *A. cassiae*-treated plants, peroxidase was elevated above that in control tissue at all sample times resulting in a 1.5 -fold increase above control in light-grown tissue and a 2- to 3-fold increase in dark-grown tissue over 48–96 h. Differences in leucine aminopeptidase activity in control versus *A. cassiae*-treated tissues were not significant until 48–96 h, when activity was inhibited in fungus-treated tissues by about 32% in light-grown tissue and 27% in dark-grown tissue after 96 h. Proteolytic activity on benzoyl-arginine-*p*-nitroanilide was not significantly different in treated versus control tissue in either light or dark over the time course. Pectinase activity increased in treated tissues at all time points as early as 16 h after spore application in light- or dark-grown plants. The greatest increases were 1.5-fold above control levels in light-grown plants (40–64 h) and 2-fold in plants grown in darkness (72–96 h). Data suggests that peroxidase may be involved as defense mechanism of sicklepod when challenged by *A. cassia* and that this mechanism is operative in young seedlings under both light and dark growth conditions. Differential proteolytic activity responses on these two substrates suggests the presence of two different enzymes. Increased pectinase activity during pathogenesis suggests that *A. cassiae*-sicklepod interaction results in an infectivity mechanism to degrade pectic polymers important to sicklepod cell wall integrity. These studies provide important information on some biochemical interactions that may be useful for improvements to biological weed control programs utilizing plant pathogens. Such information may also be useful in genetic selection and manipulation of pathogens for weed control.

## 1. Introduction

Over the past several decades many plant pathogens have been reported to act as biocontrol agents of important weeds, and numerous research and review papers have been published on this subject as summarized in selected books [1,2] and reviews [3,4,5,6,7,8,9,10,11,12,13]. Aside from reports on the isolation, formulation, efficacy, host range, etc. of these bioherbicidal pathogens, little or no information is available concerning the biochemical mechanisms related to host plant (weed) resistance or pathogen infectivity in these interactions. Various enzymes have been implicated in host plant resistance to disease. Among these are peroxidase, chitinase, glucanase, esterase, protease, glucosidase, and NADPH oxidase and several enzymes of secondary plant metabolism, including phenylalanine ammonia lyase, peroxidase, and polyphenoloxidase [14]. Most studies on plant enzymatic resistance to pathogen attack have been based on pathogen-crop plant responses and the results indicate the lack of a universal resistance mechanism(s). Hence, there is a great need to examine resistance mechanisms in pathogen/weed situations if the full potential of such interactions for biological weed control are to be realized.

Fungi from the genus, *Alternaria* represent a significant number of microorganisms studied as mycoherbicides (bioherbicides) and many produce identified metabolites that exhibit biological activity, including phytotoxicity [13]. *Alternaria* species have exhibited bioherbicidal activity on a wide variety of diverse weeds. Selected examples include: *Alternaria alternata* as a bioherbicidal against baryardgrass (*Echinochola crus-galli*) [15], *Alternaria alternata* to control pigweed (*Amaranthus retroflexus*) [16], *Alternaria helianthi* was bioherbicidal against cocklebur (*Xanthium strumarium*) [17], *Alternaria alternata* f. sp. *sphenocleae* caused leaf blight on gooseweed (*Sphenoclea zeylanica*) [18], *Alternaria eichorniae* had bioherbicidal activity on water hyacinth (*Eichhornia crassipes*) [19] and *Alternaria cassiae* could control sicklepod (*Senna obtusifolia*), showy crotolaria (*Crotolaria spectabilis*) and coffee senna (*Senna occidentalis*) [20].

*Alternaria cassiae* is a fungal plant pathogen with high efficacy for controlling its economically important host plant, the weed, sicklepod (*Senna obtusifolia*) [20,21,22,23]. Biochemical studies on this pathogen/weed interaction and on this fungus and a closely related plant showed that phenylalanine ammonia lyase activity and soluble phenolic compound levels of sicklepod seedlings treated with *A. cassiae* were substantially increased above control levels [24,25], suggesting that secondary plant metabolic processes were involved as a defense. However, the overall biochemical understanding of the mode of action of this fungus and defense mechanisms of this host weed are woefully lacking. The objective of this study was to examine several enzymes for their possible involvement in the host plant resistance or infectivity mechanisms operative in this weed/pathogen relationship. Peroxidase, proteolytic, and pectinolytic activities (enzymes implicated in defense and infectivity mechanisms) were examined. Since light can influence plant growth, infectivity, and the activity of some enzymes, these enzymes were studied in sicklepod seedlings inoculated with *A. cassiae* and grown under controlled conditions, either in continuous light or in continuous darkness over a 96-h time course.

## 2. Materials and Methods

### 2.1. Sources of Plant Tissue and Production of A. cassiae Spores

**Figure 1:**
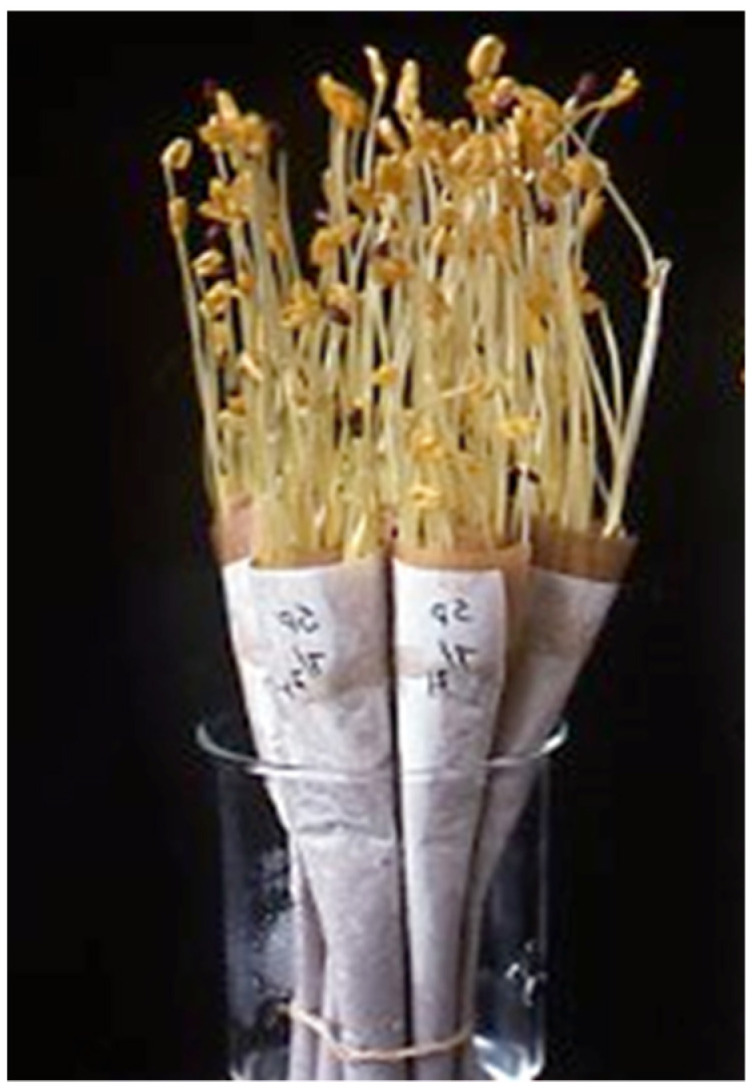
Sicklepod seedlings produced by germinating seeds in paper toweling and grown in continuous darkness.

*A. cassiae*, originally isolated from sicklepod (*Senna obtusifolia*), was cultured on a soybean flour-corn meal media and conidia (spores) were collected as described previously [22,26]. Sicklepod seeds were collected from mature plants grown at the USDA-ARS, Crop Production Systems Research Unit Research Farm in Stoneville, MS. After mechanical scarification, seeds were germinated and grown hydroponically in paper towel cylinders in 2 mM CaSO_4_ for 4 days in continuous darkness as described elsewhere [27] (Figure 1). Uniform seedlings were selected for treatment and *A. cassiae* spores (10^5^ spores mL^−1^ in a 1:1 H_2_O:soybean oil emulsion) were then applied with a small artist’s brush to the cotyledons and upper stems. Untreated seedlings received brush applications of the water: soybean oil emulsion. The pathogen-treated and untreated seedlings were then placed in a dew chamber (21–23 °C) for 16 h in darkness to facilitate spore germination and infectivity. Seedlings were then removed and placed in either the continuous darkness or continuous white light (190–200 uE m^−1^ s^−2^) at 21–23 °C regime and observations and assays were made at various time points over up to 96 h after inoculation.

### 2.2. Extraction and Enzyme Assays

At various points over a 96-h time course, sicklepod cotyledon and stem tissue samples were weighed and homogenized in 0.05 M phosphate buffer, pH 7.5 using hand-held glass homogenizers at 0–4 °C. The homogenates were clarified by centrifugation at 20,000× *g*, 15 min, followed by 30,000× *g* for 15 min. Supernatants of these extracts were assayed for enzyme activities immediately.

Peroxidase was assayed spectrophotometrically by monitoring increased absorbance at A_470_ nm resulting from the production of tetra-guaiacol from guaiacol + H_2_O_2_ [28]. Assays were performed at 25 °C in a UV/visible light spectrophotometer (Agilent 8453 UV-visible Spectroscopy System). Each assay in a total of 3.0 mL, contained 0.1 M phosphate buffer (pH 7.0), 50 µL of 20 mM guaiacol, 50 µL of 35 mM H_2_O_2_, deionized water and enzyme extract. Peroxidase activity was expressed as the change in absorbance g tissue fresh weight^−1^·min^−1^. 

Proteolytic activity was measured using two chromogenic substrates, leucine *p*-nitroanilide (leucine aminopeptidase) and *N*-α-benzoyl-L-arginine-*p*-nitroanilide (BAPA) a substrate developed for the spectrophotometric assay of the protease, trypsin [29]. Enzyme action on either of these substrates yields the product, *p*-nitroaniline that was monitored as an increase absorbance at A_410_ nm. Relative enzyme activities (▲A_410_) for leucine aminopeptidase and BAPAase were calculated and expressed on a per gram fresh weight basis.

Pectinase activity was determined by monitoring decreased absorbance at A_620_ nm which occurs as galacturonic acid methylesters are enzymatically formed from pectin + bromothymol + bromothymol blue (pH indicator) [30]. Relative enzyme activities (▲A_620_) were calculated and expressed on a g plant fresh weight^−1^ basis.

Each homogenized extract consisted of 4–6 stem and cotyledon tissue parts and each assay at each time point was performed in triplicate. The experiment was repeated in time. Error bars are presented as ± standard error.

## 3. Results

### 3.1. Germination, Growth, and Infection of A. cassia Spores

**Figure 2:**
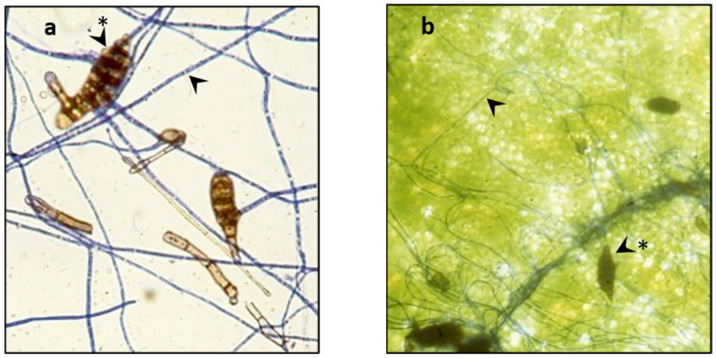
Photomicrographs of *A. cassiae* spores and mycelium (stained with lactophenol blue). Micrograph (**a**) = spores germinated on a microscope slide in soybean oil:H_2_O (1:1) and (**b**) = spores with mycelium on sicklepod cotyledon. Arrowheads point to mycelium; arrowheads-asterisk point to spores.

Mature *A. cassiae* spores began to germinate on cotyledonary and stem surfaces after 2–3 h, followed by infectivity. Figure 2a depicts germinated spores and mycelium grown 24 h on a microscope slide after staining with lactophenol blue. After 24 h, mycelial development on sicklepod cotyledons was observed, but very minimal damage was discernible (Figure 2b). Plant tissue injury increased with time and at 96-h severe necrosis and some stem collapse were evident on both light- and dark-grown plants. 

### 3.2. Peroxidase Activity

**Figure 3:**
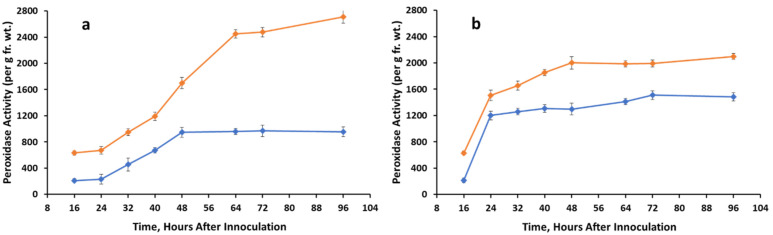
Peroxidase activity extracted from control (un-inoculated) and *A. cassiae* inoculated sicklepod seedlings at various time points. Graph (**a**) = dark growth conditions; graph (**b**) = light growth conditions. 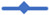 Control, 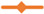 *A. cassiae*.

Peroxidase activity generally increased with time in untreated seedlings in both dark (Figure 3a) and light (Figure 3b), but the effect was greater in the light. Peroxidase activity of treated seedlings increased above control levels after 17 h (dew and total darkness). This trend continued during light or dark growth resulting in a 1.5-fold increase above control in light and a 2- to 3-fold increase in the dark over 48–96 h. 

### 3.3. Proteolytic Activity of Two Substrates

**Figure 4:**
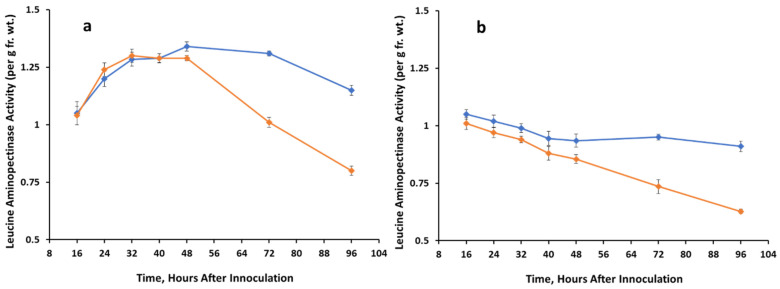
Leucine aminopeptidase activity extracted from control (un-inoculated) and *A. cassiae* sicklepod seedlings at various time points. Graph (**a**) = dark growth conditions; graph (**b**) = light growth conditions. 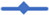 Control, 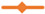 *A. cassiae*.

**Figure 5:**
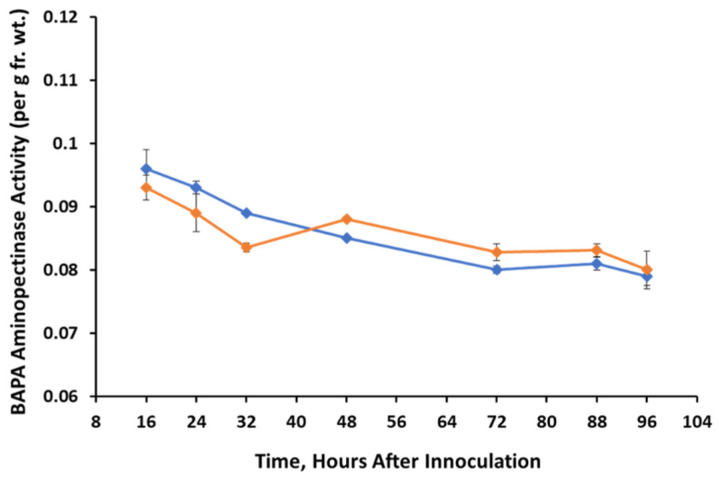
BAPA aminopeptidase activity extracted from control (un-inoculated) and *A. cassiae* inoculated sicklepod seedlings at various time points in seedlings grown under light growth conditions. 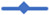 Control, 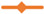 *A. cassiae*.

Differences in leucine aminopeptidase activity in control vs treated tissues were not significant until 48-96 h, when activity was inhibited in treated tissues. Inhibition was about 27% in the dark (Figure 4a) and 32% in the light (Figure 4b) after 96 h. Peptidase activity of BAPAase was not statistically significantly different in treated vs control tissue in either light (Figure 5) or dark (data not shown) over the 96-h time course. 

### 3.4. Pectinolytic Activity

**Figure 6:**
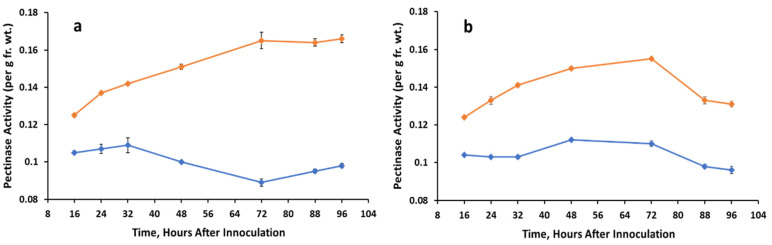
Pectinase activity extracted from control (un-inoculated) and *A. cassiae* inoculated sicklepod seedlings at various time points. Graph (**a**) = dark growth conditions; graph (**b**) = light growth conditions. 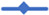 Control, 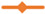 *A. cassiae*.

Pectinase activity increased in treated tissues at all time points beginning as early as 6 h after spore application in plants grown in darkness or light (Figure 6a,b). The trend continued in seedlings exposed to continuous light after 16 h (Figure 6b) and in those remaining in continuous darkness (Figure 6a). Greatest increases were 2-fold in the dark (72–96 h) and 1.5-fold in the light (40–64 h). 

## 4. Discussion

These results suggest that elevated peroxidase activity is involved in a defense mechanism of sicklepod when challenged by *A. cassiae*. This mechanism is operative in young seedlings under both light and dark growth conditions. Previously, studies of this fungus:weed interaction suggested that an important enzyme of secondary plant metabolism, phenylalanine ammonia lyase, was also involved in defense processes during infection [25]. An early and important occurrence in plant defense is an oxidative burst (production of reactive oxygen species including H_2_O_2_) [31] and peroxidases have been implicated in this process [31,32,33]. Peroxidases are also implicated in a range of defense-related processes, including the hypersensitive response, lignification and suberization and these enzymes are classified as pathogenesis-proteins. In many instances their activities and/or their gene expressions are induced in host plant tissues by pathogen (fungi and other microorganisms) infection [34,35]. Peroxidases are also involved in ethylene production and this plant hormone is associated with pathogenesis processes [36]. 

Proteases are endopeptidases that preferentially hydrolyze internal peptide bonds in polypeptide chains [37]. Many plant proteases may function in the defense pathway. For example, several pathogenesis-related proteins have been identified as proteases that accumulate in the apoplast after pathogen infection in tomato [38]. Proteolytic activity is also involved in modifying proteinaceous effectors delivered into host cells [39]. Proteases may also be involved in processing other virulence effectors delivered by pathogens. Several genes involved in the ubiquitination process have been identified as important in protein degradation irrelated to activation of the defense response [40]. Proteolytic activity may also be involved in the activation of defense regulators [41]. Aminopeptidase activity profiles using two substrates (BAPA and leucine *p*-nitroanilide) in these studies suggests the presence of two different enzymes. It is also possible that *A. cassiae* may be producing a specific peptidase inhibitor or perhaps a phytotoxin. Many compounds with diverse chemistries have been isolated from *Alternaria* spp., such as alternaric acid, alternariol, altheichin, tenuazonic acid, etc. [13]. Our strain of *A. cassiae* may also produce specific compounds that could differentially inhibit proteolytic enzymes, but the isolation and identification of such compounds was beyond the scope of this study. *Phytophthora infestans*, the oomycete species that causes late blight disease of potato and tomato, secretes an extracellular protease inhibitor (EPI1), which inhibited proteolytic activity of P69B [42]. Similarly, phytotoxins from the fungus, *Guanomyces polythrix* had inhibitory effects on the calmodulin-dependent activity of cAMP phosphodiesterase and NAD-kinase [43]. Alternatively, in the present studies, leucine aminopeptidase may be more sensitive than BAPAase to soluble phenolic compounds or other constituents released during plant cell injury and tissue maceration. Recent studies have shown that a growing list of pathogen-encoded effectors function as proteases that are secreted into plant cells to modify host proteins. In addition, several plant proteases have been found to function in activation of the defense mechanism. These findings reveal that post-translational modification of host proteins through proteolytic processing is a widely used mechanism in regulating the plant defense response.

Increased pectinase activity during pathogenesis may suggest that *A. cassiae* produces this enzyme as an infectivity mechanism to degrade pectic polymers important to sicklepod cell wall integrity. Phytopathic fungi and bacteria induce pectinase activity early during infection processes, for example during penetration [44]. However, plants can also increase pectinolytic activity in response to pathogen infection and this complex phenomenon is dependent on the specific plant: pathogen interaction [45].

## 5. Conclusions

In the present study, the activities of several enzymes involved in pathogen: plant defense were examined in an important weed: pathogen interaction. Novel information was gleaned that will provide the impetus for in-depth research approaches utilizing molecular biology techniques. Even though *Alternaria* spp. (and our strain of *A. cassiae* used here) may produce phytotoxins [13], the role of such compounds in pathogenesis has not yet been defined. Future research in these areas will improve our understanding of the mechanisms involved in the use of fungi as bioherbicidal agents of weeds.

## Data Availability

Not applicable.
